# Fabrication of Highly Crosslinked Gelatin Hydrogel and Its Influence on Chondrocyte Proliferation and Phenotype

**DOI:** 10.3390/polym9080309

**Published:** 2017-07-26

**Authors:** Xiaomeng Li, Jing Zhang, Naoki Kawazoe, Guoping Chen

**Affiliations:** 1 Research Center for Functional Materials, National Institute for Materials Science, 1-1 Namiki, Tsukuba, Ibaraki 305-0044, Japan; LI.Xiaomeng@nims.go.jp (X.L.); ZHANG.Jing@nims.go.jp (J.Z.); KAWAZOE.Naoki@nims.go.jp (N.K.); 2 Department of Materials Science and Engineering, Graduate School of Pure and Applied Sciences, University of Tsukuba, 1-1-1 Tennodai, Tsukuba, Ibaraki 305-8577, Japan

**Keywords:** gelatin, double modification, crosslinking density, chondrocyte, tissue engineering

## Abstract

Gelatin methacrylate (GelMA) hydrogels have been widely studied for biomedical applications, such as tissue engineering and drug delivery, because of their good biocompatibility and injectability. However, the quick degradation and low mechanical property of GelMA hydrogels need to be improved for further applications, especially for long-term implantation. In this study, a sequential double modification of gelatin was used to achieve high density of photocrosslinkable double bonds in gelatin derivatives. The amino groups in gelatin were first reacted with methacrylic anhydride. After this, the hydroxyl and carboxyl groups in gelatin were reacted with glycidyl methacrylate to obtain the double modified gelatin macromer. The double modified gelatin macromer was used to prepare gelatin hydrogels with high crosslinking density. The hydrogels exhibited high storage modulus and low degradation. Culture of bovine articular chondrocytes in the gelatin hydrogels showed that chondrocytes had round morphology and maintained a cartilaginous phenotype while cell proliferation was hampered. This method for increasing crosslinking density should be useful for preparation of stable hydrogels for cartilage tissue engineering.

## 1. Introduction

Cartilage tissue engineering has drawn a lot of attention due to the limited self-repairing capacity of avascular cartilage. One crucial step of cartilage tissue engineering is in vitro expansion of chondrocytes, during which chondrocytes lose part of their chondrogenic phenotype due to the differences between the two-dimensional (2D) expansion culture microenvironment and the in vivo three-dimensional (3D) microenvironment. The loss of expression of cartilaginous matrices such as collagen type II and aggrecan is a process known as de-differentiation. De-differentiated chondrocytes should be re-differentiated in order to express cartilaginous matrices for functional cartilage tissue engineering [1]. Hydrogels have been frequently used for the 3D culturing of chondrocytes for re-differentiation and maintenance of chondrogenic phenotypes and for chondrogenic differentiation of stem cells [2,3,4]. Hydrogels are hydrated polymers with 3D network structures that are similar to the microenvironments surrounding chondrocytes in vivo [5]. Many reports have shown the re-differentiation of de-differentiated chondrocytes in hydrogels [6]. Among the hydrogels, gelatin hydrogels have many advantages such as good cell adhesion (RGD peptide), good biocompatibility and easy modification [7,8,9,10,11].

Gelatin is usually modified with methacrylate groups for gelatin hydrogel preparation. The main limitations of GelMA hydrogel for tissue engineering, especially for the tissues requiring extensive load-bearing properties, are its poor mechanical properties and short degradation time [12,13]. The mechanical properties and degradation of hydrogels are related to hydrogel network crosslinking density [14]. The compressive modulus increases as the crosslinking density increases [15]. Altering precursor macromer concentration has been reported to modulate crosslinking density of hydrogels [16]. However, altering macromer concentration cannot decouple the influence of hydrogel matrix concentration. It is particularly important for gelatin hydrogel because gelatin holds RGD sequences and different gelatin concentrations may result in different densities of RGD. Other methods to increase crosslinking density include prolonging of crosslinking time [17]. However, long UV irradiation time may decrease cell viability. Incorporation of more photo-reactive double bonds to increase the ratio of photocrosslinkable bonds to photo-initiator during hydrogel preparation has been proposed as an attractive method to increase crosslinking density because a high ratio of photocrosslinkable bonds to photo-initiator can protect cells from free radical attack and therefore lead to high cell viability [18].

For the methacrylation of gelatin molecules, amino groups are usually used for reaction with methacrylate groups. However, the number of amino groups in each gelatin molecule is limited. There are many hydroxyl and carboxyl groups in gelatin molecules. Therefore, usage of these functional groups for further methacrylation of gelatin is desirable to increase the crosslinking density of gelatin hydrogels.

In this study, a sequential double modification of amino, hydroxyl, and carboxyl groups of gelatin molecules was used to introduce more photocrosslinkable methacrylate groups in gelatin for the preparation of gelatin hydrogels with a high crosslinking density. The elastic property and enzymatic degradation rate of the hydrogel were evaluated. The gelatin hydrogels were used for 3D culturing of chondrocytes, and their effects on cell proliferation and expression of cartilaginous matrices were investigated. 

## 2. Materials and Methods

### 2.1. Synthesis of GelMA and GelMAGMA

GelMA macromer was synthesized according to a previously described method [7]. Briefly, 5 g gelatin (type A, 300 bloom, Sigma-Aldrich, St. Louis, MO, USA) was dissolved in phosphate buffered saline (PBS) at 50 °C under stirring to obtain a 10% (*w*/*v*) gelatin solution. 5 mL methacrylic anhydride (MA; Sigma-Aldrich, St. Louis, MO, USA) was added into the gelatin solution at a rate of 0.5 mL/min while stirring at 50 °C to prepare GelMA. After reaction in the dark for 3 h, the product was diluted with five-fold warm PBS (50 °C) and then dialyzed against Milli-Q water for seven days at 40 °C using a dialysis membrane (12–14 kD molecular weight cut-off, Spectrum Laboratories Inc., Rancho Dominguez, CA, USA) to remove salts and excess free MA. The GelMA foam was obtained after freeze-drying for two days.

Glycidyl methacrylate-modified GelMA (GelMAGMA) macromer was synthesized by modifying the GelMA macromer with glycidyl methacrylate [19]. After dissolving 2.5 g of GelMA macromer in Milli-Q water (2%, *w*/*v*), the pH of the solution was adjusted to 3.5 with 1 M HCl (Wako, Osaka, Japan). After this, 5 mL of glycidyl methacrylate (GMA; Sigma-Aldrich, St. Louis, MO, USA) was added into the solution at a rate of 0.5 mL/min [20]. The reaction was conducted at 50 °C for 24 h and the product was purified by dialysis against Milli-Q water with the above-mentioned dialysis membrane at 40 °C for seven days. The purified product was freeze-dried and stored at −20 °C for further use.

### 2.2. ^1^H Nuclear Magnetic Resonance (NMR)

The degree of functionalization was studied by using ^1^H NMR according to a previously reported method [21]. ^1^H NMR spectra were collected by using an NMR spectrometer (AL300; JEOL, Tokyo, Japan) with a single axis gradient inverse probe at a frequency of 300 MHz. Before the measurement, 30 mg of GelMA and GelMAGMA macromers were respectively dissolved into 1 mL deuterium oxide containing 0.05% (*w*/*v*) 3-(trimethylsilyl)propionic-2,2,3,3-d4 acid sodium salt (Sigma-Aldrich, St. Louis, MO, USA) for calibration at 40 °C.

### 2.3. Preparation of GelMA and GelMAGMA Hydrogels

GelMA and GelMAGMA solutions were prepared by dissolving the respective macromers (10%, *w*/*v*) and photoinitiator, 2-hydroxy-1-(4-(hydroxyethoxy) phenyl)-2-methyl-1-propanone (I 2959, Sigma-Aldrich, St. Louis, MO, USA), (0.5%, *w*/*v*) in PBS solution at 50 °C. The above solutions were sterilized by filtration through a 0.22 µm filter (Millex-GV, Carrigtohill, Cork, Ireland). After sterilization, the solution was pipetted between two sterile quartz glass coverslips separated by a sterile silicone membrane frame with a 1.5 mm thickness (KOKEN Co., Ltd., Tokyo, Japan) and exposed to UV light (CL-1000, Funakoshi Co., Ltd., Tokyo, Japan) for 5 min. After cross-linking, the hydrogels were punched into disks with a 10 mm biopsy punch (Acu-Punch, Acuderm Inc., Fort Lauderdale, FL, USA) for rheological testing and a 6 mm biopsy punch (Kai Medical, Gifu, Japan) for the other experiments.

### 2.4. Rheological Measurements

The storage modulus of GelMA and GelMAGMA hydrogel disks (10 mm of diameter) were measured by an MCR301 Rheometer (Anton Paar, Ostfildern, Germany) equipped with 10 mm parallel plates. The temperature was set at 37 °C, and the samples were balanced for 3 min before the start of testing. The storage moduli of GelMA and GelMAGMA hydrogels were measured by an oscillatory shear deformation at a constant frequency (1 Hz) and a constant shear strain (5%) [21]. Three samples were tested to calculate means and standard deviations.

### 2.5. Swelling Ratio and Enzymatic Degradation of the Hydrogels

GelMA and GelMAGMA hydrogel disks (6 mm of diameter) were swollen in PBS for 24 h to reach swelling equilibrium. The samples were blotted with a KimWipe paper to remove the residual liquid and weighed to obtain the equilibrium wet weight. The dry weight was measured after freeze-drying. The swelling ratio was calculated according to a previous report [22]. Three samples were used for the measurement to calculate means and standard deviations.

The swollen hydrogel disks were immersed in 5 mL of PBS containing 10 units·mL^−1^ of collagenase I (Worthington Biochemical, Lakewood, NJ, USA) and incubated at 37 °C in an orbital shaker at a shaking speed of 100 rpm. At the designated time points, the hydrogel disks were taken out and weighed after the removal of the excess solution by blotting. The degradation degree of the hydrogels was calculated by normalizing the residual hydrogel wet weight to the initial wet weight. Five samples were used at every time point for the measurement to calculate means and standard deviations.

### 2.6. Chondrocytes Isolation and Culture In Vitro

Bovine articular chondrocytes (BACs) were isolated from articular cartilage from the knees of a calf according to previously reported protocol [23]. Briefly, the articular cartilage tissue was minced into small pieces using a sterile surgery scalpel. The minced tissue pieces were digested with a 0.2% *w*/*v* collagenase type II (Worthington Biochemical, Lakewood, NJ, USA) aqueous solution overnight at 37 °C with shaking. The digestion solution was filtered through a sterile nylon mesh with a 70 µm mesh size to remove any undigested fragments. The isolated primary chondrocytes were collected by centrifugation and cultured in 75 cm^2^ tissue culture flasks in Dulbecco’s Modified Eagle Medium (D6546; Sigma-Aldrich, St. Louis, MO, USA) supplemented with 10% fetal bovine serum, 4500 mg L^−1^ glucose, 4 mM glutamine, 100 U·mL^−1^ penicillin, 100 µg mL^−1^ streptomycin, 0.1 mM nonessential amino acids, 0.4 mM proline, 1 mM sodium pyruvate, and 50 µg·mL^−1^ ascorbic acid at 37 °C and 5% CO_2_. The culture medium was refreshed every three days. The cells were subcultured after reaching confluence. The chondrocytes at passage 2 were used in the following experiments. The cells were detached with a trypsin/EDTA solution, collected by centrifugation, counted with a hemocytometer, and re-suspended in the mixture solution of GelMA or GelMAGMA macromer (10%, *w*/*v*) and photo-initiator (0.5%, *w*/*v*) sterilized by 0.22 µm filter. The cell suspension solution was added between two quartz glass coverslips separated by a 1.5 mm-thick silicone membrane frame and exposed to UV light to prepare cell-laden GelMA or GelMAGMA hydrogel as described in Section 2.3. The quartz glass and silicone frame were sterilized by 70% ethanol and washed with PBS. The cell-laden hydrogels were punched into disks with a 6 mm biopsy punch. Before culturing, all the samples were washed twice to remove the photo-initiator by immersing in culture medium for 30 min. The cell-laden hydrogel disks were transferred to cell culture T-flasks for dynamic cell culture under shaking (60 rpm). The culture medium was changed every two days. All the procedures were conducted under sterile conditions on a clean bench. The UV crosslinking machine was sterilized by 70% ethanol and moved into the clean bench before UV crosslinking. As a two-dimensional culture control, the passage 2 chondrocytes were seeded in 24-well tissue culture plates (TCP) and cultured with the same medium as that used for three-dimensional culture in the hydrogels.

### 2.7. Cell Viability Assay

Live/dead staining was performed to evaluate cell viability of chondrocytes in the hydrogels by using Cellstain Double Staining Kit (Dojindo Laboratories, Tokyo, Japan). After UV crosslinking and 14 days of in vitro culture, the cell-laden hydrogel disks were washed with PBS three times and incubated with serum-free medium containing calcein-AM (2 µM) and propidium iodide (4 µM) at 37 °C for 15 min. The stained cells were observed using a confocal microscope (Zeiss LSM 510 Meta).

### 2.8. In Vivo Implantation

All the animal experiment procedures were approved by the Animal Experiments Committee of the National Institute for Materials Science and the experiment was conducted according to the committee guidelines. After the cell-laden hydrogel disks were cultured in the culture medium under shaking for one day, they were subcutaneously implanted in the dorsa of athymic nude mice. After six weeks of implantation, the mice were euthanized and the implants were harvested for further study. 

### 2.9. Quantification of DNA and Sulfated Glycosaminoglycan (sGAG)

DNA amount and sGAG content in the hydrogel disks were quantified after six weeks of implantation. The harvested cell-laden hydrogel disk implants were washed with PBS three times and freeze-dried. Each of the freeze-dried implants was digested by 500 µL papain solution (Sigma-Aldrich, St. Louis, MO, USA), which was prepared by dissolving papain at a concentration of 400 mg/mL in 0.1 M PBS (pH 6.0) containing 5 mM cysteine hydrochloride and 5 mM ethylenediaminetetraacetic acid (EDTA). 5 µL of the papain digestion solution was used to measure the DNA amount with Hoechst 33258 dye (Sigma-Aldrich, St. Louis, MO, USA). The fluorescence intensity was read with an FP-6500 spectrofluorometer (JASCO, Tokyo, Japan) at an excitation/emission wavelength of 360 and 460 nm. The sGAG content in each digestion solution was measured by using a BlyscanTM Glycosaminoglycan Assay Kit (Biocolor Ltd., County Antrim, UK). Four samples in each group were used for the measurement to calculate means and standard deviations.

### 2.10. Histological and Immunohistochemical Staining

The harvested cell-laden hydrogel disk implants were washed three times with PBS and fixed with 10% neutral buffer formalin (Wako, Osaka, Japan) at room temperature for two days. After that, the implants were dehydrated in a series of ethanol solutions with increasing ethanol concentration from 70% to 99.5%, embedded in paraffin, and sectioned with a microtome (Leica RM2245; Wetzlar, Germany) to obtain cross-sections having a 7 µm thickness. The cross-sections were then de-paraffinized and stained with hematoxylin and eosin (HE) for cell morphology; and safranin O/fast green and alcian blue for glycosaminoglycan. Briefly, the de-paraffinized sections were immersed in hematoxylin solution for 10 min, and then washed with tap water for 10 min before immersing in eosin solution for 3 min. For safranin O/fast green staining, the de-paraffinized sections were stained with 0.02% aqueous solution of fast green for 1 min, followed by washing with tap water, immersion in 1% acetic acid aqueous solution for 15 s and immersion in 0.1% safranin O aqueous solution for 10 min. The alcian blue staining was conducted by immersing the de-paraffinized sections in alcian blue aqueous solution for 30 min. The immunohistochemical staining of collagen type II and collagen type I was performed according to a previous report [24]. Briefly, the de-paraffinized sections were incubated with proteinase K (Dako Corp., Carpinteria, CA, USA) for 10 min for antigen retrieval, and then incubated with peroxidase blocking solution (Dako) for 5 min and 10% goat serum solution (Dako) for 30 min. Next, the sections were incubated with the first antibodies for 2 h at room temperature, followed by incubation with the peroxidase-labeled polymer-conjugated second antibodies (DakoCytomation Envision+, Dako, Carpinteria, CA, USA) for another 30 min at room temperature. The first antibodies were rabbit polyclonal anti-collagen type II at a 1:100 working dilution (AB746; Millipore, Schwalbach, Germany) and rabbit monoclonal anti-collagen type I at a 1:100 working dilution (AB138492; Abcam, Cambridge, MA, USA). The second antibody was HRP-labeled polymer conjugated secondary antibody (anti-rabbit; Dako, Carpinteria, CA, USA). The sections were finally incubated with 3,3′-diaminobenzidine (DAB; Dako) for 10 min to develop color. The chondrocytes cultured in 24-well tissue culture plates were stained with the same protocols after reaching confluence by five days of culturing. The stained samples were observed under an optical microscope.

### 2.11. Real-Time Polymerase Chain Reaction (PCR) Analysis

Expression of genes encoding collagen type II, aggrecan, Sox 9, and collagen type I were analyzed by a real-time PCR [25]. The cell-laden hydrogel disk implants were washed with PBS three times, frozen in liquid nitrogen, and crushed into powder by an electric crusher. The powder samples were dissolved in Sepasol solution (1 mL per sample; Nacalai tesque, Kyoto, Japan) to isolate the RNA. The RNA content in each sample was quantified, after which the RNA was converted to cDNA by MuLV reverse transcriptase (Applied Biosystems, Foster City, CA, USA). Real-time PCR was used to amplify glyceraldehyde-3-phosphate dehydrogenase (Gapdh), type II collagen (Col-II), aggrecan (Acan), Sox 9 and type I collagen (Col-I) by using a 7500 real-time PCR System (Applied Belgium, Foster City, CA, USA). The expression level of Gapdh, a housekeeping gene, was used as an endogenous control. The relative expression of the target gene was calculated by using the 2^−ΔΔ*C*T^ formula and passage 2 chondrocytes as a reference. The primer and probe sequences were the same as those used in the previous study. Four samples in each group were used for the measurement to calculate means and standard deviations.

### 2.12. Statistical Analysis

All data were reported as the mean ± standard deviation (SD). Statistical analysis was performed using a one-way ANOVA analysis to evaluate the significance of the experimental data. Statistical differences were considered significant if *p* < 0.05. (*), (**) and (***) indicated *p* < 0.05, *p* < 0.01 and *p* < 0.001, respectively. All statistical analyses were executed by using KyPlot 2.0 beta 15 (1997–2001 Koichi Yoshioka).

## 3. Results and Discussion

### 3.1. Synthesis and ^1^H NMR of GelMA and GelMAGMA Macromers

**Figure 1:**
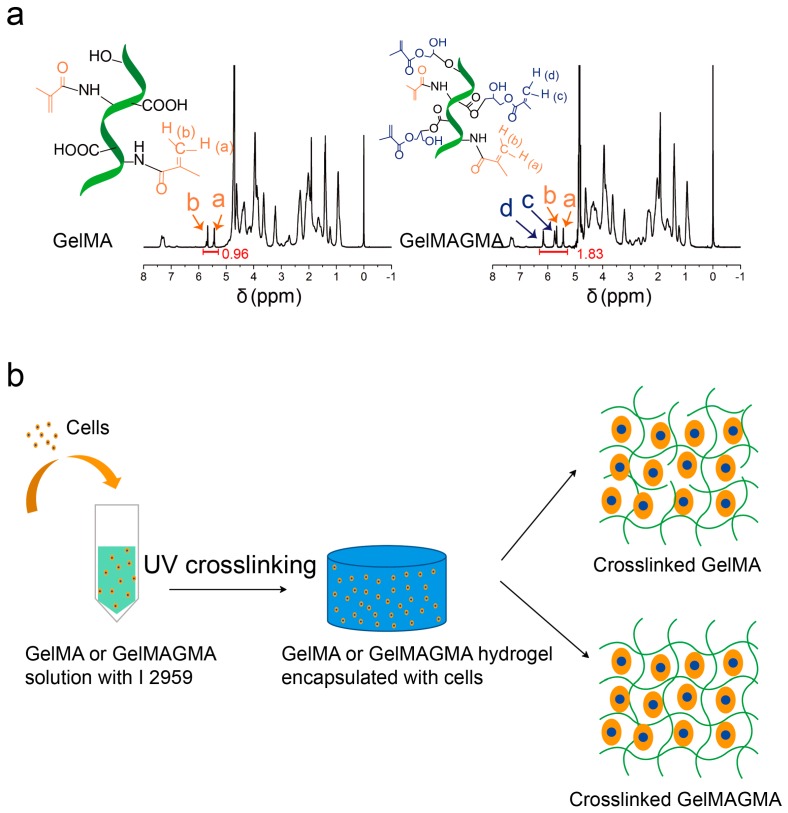
^1^H NMR spectra of GelMA and GelMAGMA macromers (**a**). Experimental scheme of GelMA and GelMAGMA hydrogels encapsulated with chondrocytes and illustration of cell-laden hydrogel networks (**b**).

Gelatin has abundant amino, hydroxyl, and carboxyl groups that can be used for modification. After the reaction of the amino groups in gelatin molecules with methacrylic anhydride, the remaining hydroxyl and carboxyl groups can be used for further modification in an acidic environment [20]. GelMA macromer was first synthesized, and then the GelMA macromer was used for the second modification to synthesize GelMAGMA. The ^1^H NMR spectra of GelMA and GelMAGMA macromers are shown in Figure 1a. The protons in methacrylate groups are indicated in the ^1^H NMR spectra. Integrated intensity of the protons in methacrylate groups of the GelMAGMA macromer (1.83) was much higher than that of the GelMA macromer (0.96). The result indicated that the methacrylate group density in the GelMAGMA macromer was higher than that in the GelMA macromer. The second modification by reaction between the hydroxyl and carboxyl groups with glycidyl methacrylate increased the photocrosslinkable double bonds in the GelMAGMA macromer, which should result in high degree of cross-linking in the GelMAGMA hydrogels. The GelMA and GelMAGMA macromers were used to prepare gelatin hydrogels having respectively low and high densities of cross-linking. Chondrocytes were suspended in the macromer solution before UV irradiation to prepare cell-laden gelatin hydrogels (Figure 1b).

### 3.2. Rheological Property of GelMA and GelMAGMA Hydrogels

**Figure 2:**
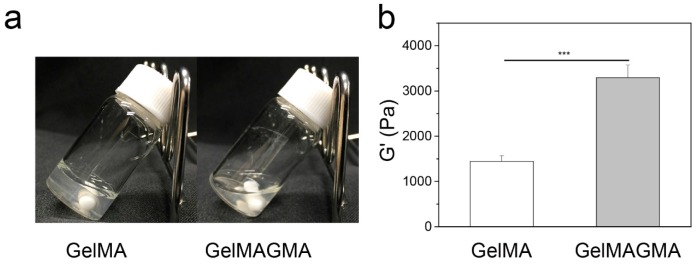
Gross appearance of GelMA and GelMAGMA solution (10%, *w*/*v*) at room temperature (**a**). The storage modulus (*G*′) of GelMA and GelMAGMA hydrogels at 37 °C with a constant deformation of 5% and a frequency of 1 Hz (**b**). The data represent means ± SD, N = 3. *** *p* < 0.005.

The GelMA solution became hydrogel at room temperature, while the GelMAGMA solution kept its solution state even at room temperature (Figure 2a), indicating a reduction of the intermolecular interaction forces of the gelatin chains by modification [21].

The storage modulus (*G*′) of the GelMA and GelMAGMA hydrogels (10%, *w*/*v*) was measured at a constant deformation of 5% and a frequency of 1 Hz (Figure 2b). The storage modulus of the GelMAGMA hydrogel was significantly higher than that of the GelMA hydrogel. This should be due to high crosslinking density and steric hindrance between polymer chains in the GelMAGMA hydrogel. It has been reported that compressive modulus of a GelMA hydrogel increases with the degree of modification [26]. Hydrogels with different modification degrees and concentrations of GelMA have been reported to exhibit a broad range of compressive moduli from 3 to 30 kPa [7]. Hydrogels with 30 kPa Young’s modulus can be formed with 15% GelMA having a modification degree of 80%. In our previous study, the compressive modulus of GelMA hydrogel could be enhanced to 30 kPa by using 10% GelMA macromer having a modification degree of 90% [9]. The GelMAGMA hydrogel in this study showed a higher storage modulus than did the GelMA hydrogel. The results indicated that the stiffness of gelatin-based hydrogel could be increased by sequential modification with methacrylic anhydride and glycidyl methacrylate without changing gelatin concentration. It has been reported that the different properties of crosslinked hydrogels—such as storage modulus, swelling ratio, and degradation rate—can be controlled by network crosslinking density [14]. It has also been reported that the mechanical property of GelMA hydrogel can be adjusted by modification degree or macromer concentration [7,9]. The sequential double modification of amino, hydroxyl, and carboxyl groups in gelatin molecules was demonstrated as an efficient way to adjust the crosslinking density and Young’s modulus of gelatin hydrogels. 

### 3.3. Swelling Behavior and Enzymatic Degradation

**Figure 3:**
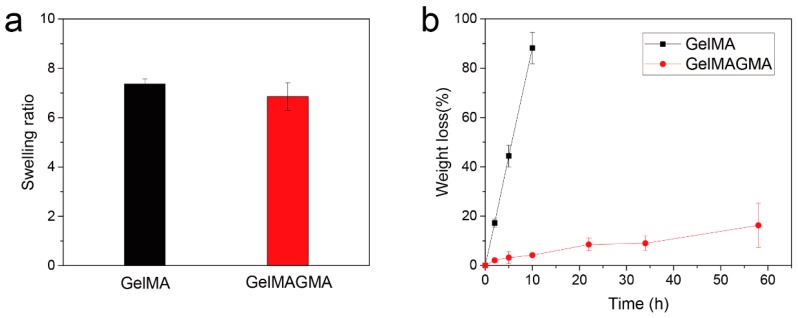
Equilibrium swelling ratio of GelMA and GelMAGMA hydrogels (**a**), the data represent means ± SD, N = 3. Enzymatic degradation of GelMA and GelMAGMA hydrogels in the presence of 10 unit mL^−1^ of collagenase type I at 37 °C with shaking (**b**), the data represent means ± SD, N = 5.

The swelling ratio of hydrogels is controlled by polymer network mesh size and polymer-solvent interaction. The swelling ratio of GelMAGMA hydrogel was slightly lower than that of GelMA hydrogel, although the difference was not significant (Figure 3a). The low swelling ratio of GelMAGMA hydrogel is most likely due to its high crosslinking density. 

Enzymatic degradation showed that the GelMAGMA hydrogel was more stable than the GelMA hydrogel (Figure 3b). The GelMA hydrogel was degraded rapidly by collagenase I. In contrast, the GelMAGMA hydrogels showed a very slow degradation rate. Gelatin hydrogels could be degraded by collagenase I easily while crosslinking inhibited its degradation. A higher crosslinking density led to a slower degradation rate. The slow degradation rate of GelMAGMA hydrogel should allow for long-term implantation.

### 3.4. Cell Viability in the Hydrogels During In Vitro Culture

**Figure 4:**
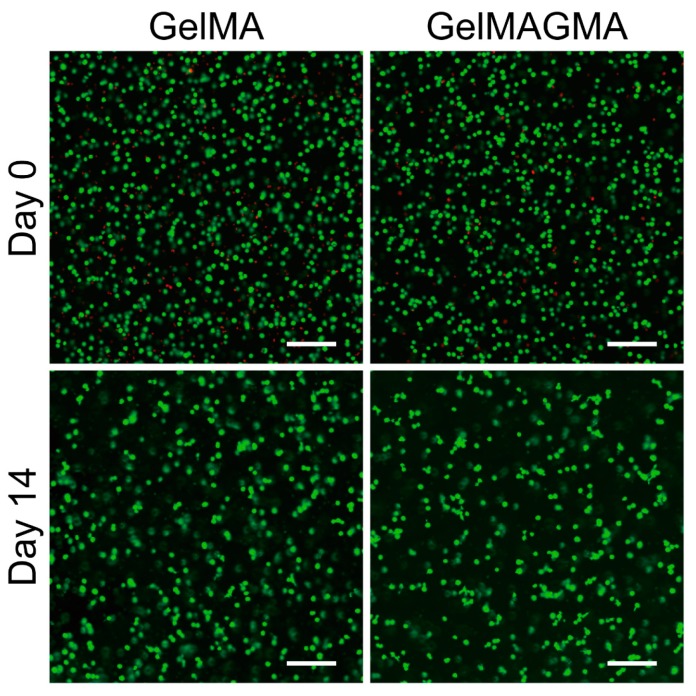
Live and dead staining of chondrocytes in GelMA and GelMAGMA hydrogels after UV crosslinking (Day 0) and after 14 days in vitro culturing (Day 14). Scale bar = 200 μm. (Green: live cells; Red: dead cells).

Live/dead staining was used to evaluate cell viability in the two kinds of hydrogels immediately after UV crosslinking (Day 0) and after two weeks of in vitro culturing (Day 14) (Figure 4). A small number of chondrocytes were detected dead after UV-initiated polymerization. After culturing for two weeks, chondrocytes in all the groups showed high cell viability. Some chondrocytes integrated to form small aggregates. Chondrocytes in the GelMA and GelMAGMA hydrogels showed similar results. The results indicated that the UV-initiated polymerization of photocrosslinkable macromers did not evidently affect cell viability and that the GelMA and GelMAGMA hydrogels were compatible for chondrocyte culturing. 

### 3.5. DNA and sGAG Quantification

**Figure 5:**
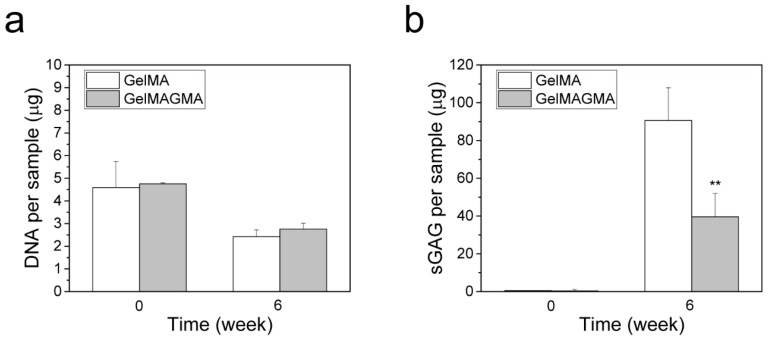
DNA amount (**a**) and sGAG content (**b**) in GelMA and GelMAGMA hydrogels before implantation (0 week) and after in vivo implantation for six weeks (6 week). Means ± SD, N = 4. ** *p* < 0.01.

DNA amount and sGAG content in the cell-laden hydrogel disk implants at the beginning (week 0) and after six weeks implantation (week 6) were measured (Figure 5). After six weeks of implantation, the DNA amount decreased in both GelMA and GelMAGMA hydrogels. However, the sGAG content increased significantly after six weeks of implantation. sGAG is one of the main components of native cartilage and is used to evaluate chondrogenic activity [27]. The decrease of DNA amount might be due to partial cell death and limited nutrient diffusion and penetration in the hydrogels. Formation of cartilaginous matrices in hydrogel has been reported to hamper nutrient diffusion and therefore affect cell proliferation during long period implantation [28]. The high sGAG content indicated the hydrogels were beneficial for secretion of cartilaginous matrices, in particular, the GelMA hydrogel.

### 3.6. Histological and Immunohistochemical Stainings

**Figure 6:**
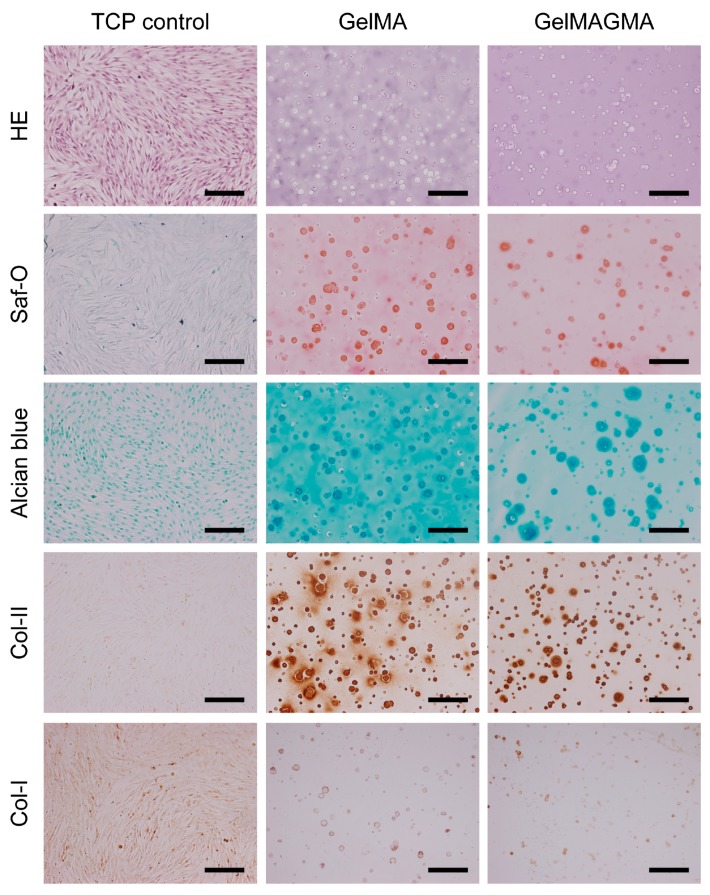
Hematoxylin and eosin (HE), safranin O (Saf-O), and alcian blue staining and immunohistochemical staining of collagen type II (Col-II) and collagen type I (Col-I) of the GelMA and GelMAGMA hydrogel implants after subcutaneous implantation for six weeks. Chondrocytes cultured in 24-well tissue culture plates (TCP) for five days were stained as a control. Scale bar = 200 μm.

HE staining showed that chondrocytes in both GelMA and GelMAGMA hydrogels had round morphology that were similar to chondrocyte morphology in native cartilage (Figure 6). It has been reported that round cell morphology, possessing weak actin cytoskeletal organization, is beneficial for chondrogenesis [29]. Partial chondrocytes aggregated to form small aggregates. Cell aggregation has been reported to be good for chondrogenic ECM secretion and chondrocyte phenotype maintenance [30,31]. Safranin O and alcian blue staining results showed that cartilaginous matrices were detected around the round cells. Immunohistochemical staining of type II collagen also demonstrated the presence of type II collagen in areas surrounding the round cells. Both GelMA and GelMAGMA hydrogels showed slight staining of type I collagen. The chondrocytes in GelMA hydrogels showed slightly stronger staining of chondrogenic matrices than did the cells in GelMAGMA hydrogels. The high crosslinking density in GelMAGMA hydrogels might inhibit the diffusion of cartilaginous matrices. It has been reported that diffusion of ECM is related to the hydrogel network structure and diffusivity decreases when the crosslinking density increases [32].

To further compare the morphology and phenotype of chondrocytes in 2D and 3D, the same chondrocytes (passage 2) were cultured in cell culture plates for five days. The chondrocytes reached confluence after five days of culturing. The chondrocytes showed elongated morphology. Safranin O and alcian blue staining showed that no cartilaginous matrices were detected. Immunohistochemical staining of type I and type II collagen showed that type I collagen was detected while no type II collagen was detected. Many studies have reported that chondrocytes change their morphology from round to spindle-like elongated shape and lose the capacity to express cartilaginous matrices during 2D expansion culturing [33,34,35].

### 3.7. Cartilaginous Gene Expression

**Figure 7:**
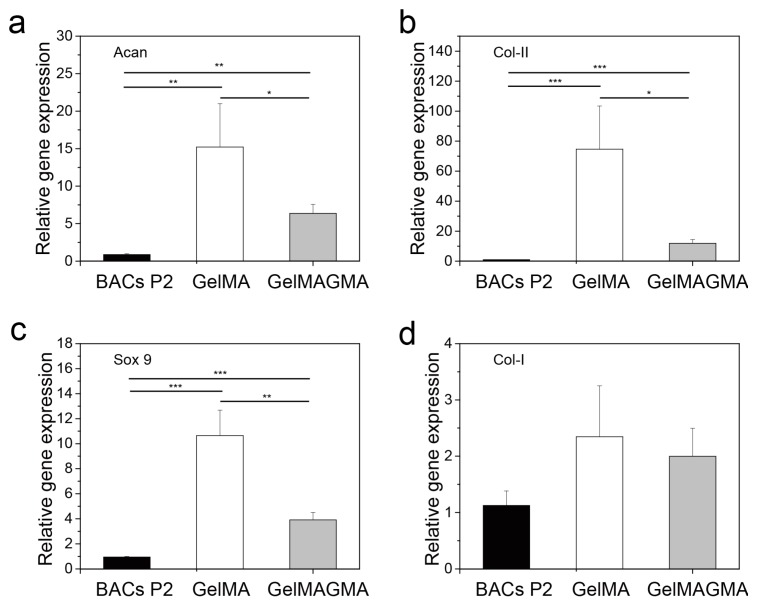
Expression of genes encoding aggrecan (**a**), collagen II (**b**), Sox 9 (**c**), and collagen I (**d**) of chondrocytes in GelMA and GelMAGMA hydrogel implants after six weeks of implantation. Data represent means ± SD, N = 3. * *p* < 0.05; ** *p* < 0.01; *** *p* < 0.005.

To further compare the influence of GelMA and GelMAGMA hydrogels on chondrocyte phenotype, gene expression of aggrecan (Figure 7a), collagen type II (Figure 7b), Sox9 (Figure 7c), and collagen type I (Figure 7d) were investigated by real-time PCR. Collagen type II and aggrecan are the two main characteristic genes related to chondrogenic differentiation [36]. Sox9 is the key transcription factor of chondrogenesis and chondrogenic differentiation [37]. The expression level of collagen type II, aggrecan, and Sox9 was significantly higher than that of chondrocytes used for cell seeding, while there was no significant difference in expression of collagen type I. The results indicated that culturing in the hydrogels upregulated cartilaginous gene expression. The hydrogels could provide a 3D microenvironment for expression of cartilaginous genes. The expression level of the cartilaginous genes in the GelMA hydrogel was significantly higher than that in the GelMAGMA hydrogel.

In this study, two types of gelatin hydrogels were prepared by modifying the amino, hydroxyl, and carboxyl groups in gelatin molecules with photocrosslinkable methacrylate groups. The first modification was conducted by reacting amino groups in gelatin molecules with methacrylic anhydride. The second modification was carried out by coupling the hydroxyl and carboxyl groups with glycidyl methacrylate. After double modification, more methacrylate groups were introduced in gelatin molecules. The hydrogels prepared from the double modified GelMAGMA macromer had higher crosslinking density and storage modulus than did the hydrogels prepared from single modified GelMA macromer. The GelMAGMA hydrogel was degraded more slowly than the GelMA hydrogel. By using the sequential double modification, gelatin hydrogels with a broad difference of storage modulus could be prepared. 

Many methods have been reported to modulate the mechanical properties of gelatin hydrogels, including the incorporation of nanoparticles, hybridization with other polymers, UV irradiation dosage, and modification degree [38,39]. The previous two methods may induce other components in the hydrogels. High dosage and long UV irradiation may induce cellular damage. Modification degree can be modulated by binding a different amount of methacrylate groups with the amino groups in gelatin molecules. However, the range of mechanical properties that can be tuned by methacrylation of amino groups is limited. The sequential double modification of the amino, hydroxyl, and carboxyl groups in gelatin broadened the tunable modification degree and mechanical property range. There are many crosslinking methods that can be used to prepare gelatin hydrogels and scaffolds for biomedical applications [40,41,42,43]. Crosslinking reagents such as carbodiimides and succinimides [43], glutaraldehyde [44], and genipin [45], and high energy irradiation [46], have been used for the crosslinking. However, these methods should be used to crosslink the hydrogels and scaffolds before cell-lading to avoid cytotoxicity. In this study, UV-initiated polymerization was used to crosslink the GelMA and GelMAGMA macromers and had no evident negative influence on cell viability.

Both GelMA and GelMAGMA hydrogels showed promotive effects for chondrocytes to keep round morphology and express cartilaginous matrices. However, the hydrogels hampered cell proliferation. The effects should be due to the 3D microenvironment in hydrogels that are beneficial for chondrogenic differentiation rather than cell proliferation. By comparison of the two gelatin hydrogels with low and high storage modulus, the GelMA hydrogel (low storage modulus) was more beneficial to cartilaginous matrices expression than the GelMAGMA hydrogel (high storage modulus). Physical cues such as stiffness and cell size have been reported to have influences on cell functions [47,48]. In our previous study, gelatin hydrogel with the highest stiffness showed the best effects on maintaining the phenotype of chondrocytes. The gelatin hydrogel with the highest stiffness in the previous study was the GelMA hydrogel in this study. Further increasing the stiffness and storage modulus had no effect on the promotion of expression of cartilaginous matrices, nor the maintenance of chondrogenic phenotype. It has been reported that gelatin-hydroxyphenylpropionic acid hydrogel with a storage modulus of 1000 Pa has the best function for cartilage regeneration. Therefore, the GelMA hydrogel should provide the optimal microenvironment for maintenance of chondrocyte phenotypes. Influence of the second modification on the bioactive groups such as the RGD motif in gelatin molecules might be another reason for the different effect on chondrocyte functions, which needs confirmation in the future. The good mechanical property and slow degradation of the GelMAGMA hydrogel should provide a variety of opportunities for incorporation of other bioactive factors or introduction of microporous structures in the hydrogels for cartilage tissue engineering.

## 4. Conclusions

Gelatin macromer with a high degree of modification was synthesized by sequential double modification of amino, hydroxyl, and carboxyl groups in gelatin molecules. The double modified GelMAGMA macromer was used to prepare chondrocyte-laden hydrogel for 3D culturing of chondrocytes. The GelMAGMA hydrogel had a higher storage modulus and slower enzymatic degradation than did the hydrogel prepared with single modified GelMA macromer. The GelMAGMA hydrogel facilitated expression of cartilaginous matrices, as well as the maintenance of cartilage phenotype, while hampering cell proliferation. The GelMAGMA hydrogel should be useful for strengthening hydrogel scaffolds with a low degradation rate for cartilage tissue engineering.

## References

[1] Chen G., Sato T., Ushida T., Hirochika R., Tateishi T. (2003). Redifferentiation of dedifferentiated bovine chondrocytes when cultured in vitro in a PLGA-collagen hybrid mesh. FEBS Lett..

[2] Yan S., Zhang X., Zhang K., Di H., Feng L., Li G., Fang J., Cui L., Chen X., Yin J. (2016). Injectable in situ forming poly (l-glutamic acid) hydrogels for cartilage tissue engineering. J. Mater. Chem. B.

[3] Deepthi S., Gafoor A.A.A., Sivashanmugam A., Nair S.V., Jayakumar R. (2016). Nanostrontium ranelate incorporated injectable hydrogel enhanced matrix production supporting chondrogenesis in vitro. J. Mater. Chem. B.

[4] Wang T., Lai J.H., Han L.-H., Tong X., Yang F. (2016). Modulating stem cell–chondrocyte interactions for cartilage repair using combinatorial extracellular matrix-containing hydrogels. J. Mater. Chem. B.

[5] Serafim A., Tucureanu C., Petre D.-G., Dragusin D.-M., Salageanu A., Van Vlierberghe S., Dubruel P., Stancu I.-C. (2014). One-pot synthesis of superabsorbent hybrid hydrogels based on methacrylamide gelatin and polyacrylamide. Effortless control of hydrogel properties through composition design. New J. Chem..

[6] Zeng L., Chen X., Zhang Q., Yu F., Li Y., Yao Y. (2015). Redifferentiation of dedifferentiated chondrocytes in a novel three-dimensional microcavitary hydrogel. J. Biomed. Mater. Res. A.

[7] Nichol J.W., Koshy S.T., Bae H., Hwang C.M., Yamanlar S., Khademhosseini A. (2010). Cell-laden microengineered gelatin methacrylate hydrogels. Biomaterials.

[8] Yue K., Trujillo-de Santiago G., Alvarez M.M., Tamayol A., Annabi N., Khademhosseini A. (2015). Synthesis, properties, and biomedical applications of gelatin methacryloyl (GelMA) hydrogels. Biomaterials.

[9] Li X., Chen S., Li J., Wang X., Zhang J., Kawazoe N., Chen G. (2016). 3D culture of chondrocytes in gelatin hydrogels with different stiffness. Polymers.

[10] Zhang J., Yang Y., Chen Y., Liu X., Guo S., Zhu L., Wang Y. (2016). An in situ phototriggered-imine-crosslink composite hydrogel for bone defect repair. J. Mater. Chem. B.

[11] Yu Y., Guo L., Wang W., Wu J., Yuan Z. (2015). Dual-peptide-modified alginate hydrogels for the promotion of angiogenesis. Sci. China Chem..

[12] Han L., Xu J., Lu X., Gan D., Wang Z., Wang K., Zhang H., Yuan H., Weng J. (2017). Biohybrid methacrylated gelatin/polyacrylamide hydrogels for cartilage repair. J. Mater. Chem. B.

[13] Wang H., Zhou L., Liao J., Tan Y., Ouyang K., Ning C., Ni G., Tan G. (2014). Cell-laden photocrosslinked GelMA–DexMA copolymer hydrogels with tunable mechanical properties for tissue engineering. J. Mater. Sci. Mater. Med..

[14] Bryant S.J., Anseth K.S. (2002). Hydrogel properties influence ECM production by chondrocytes photoencapsulated in poly (ethylene glycol) hydrogels. J. Biomed. Mater. Res. A.

[15] Metters A., Anseth K., Bowman C. (2000). Fundamental studies of a novel, biodegradable PEG-*b*-PLA hydrogel. Polymer.

[16] Wang L.-S., Du C., Toh W.S., Wan A.C., Gao S.J., Kurisawa M. (2014). Modulation of chondrocyte functions and stiffness-dependent cartilage repair using an injectable enzymatically crosslinked hydrogel with tunable mechanical properties. Biomaterials.

[17] Bian L., Hou C., Tous E., Rai R., Mauck R.L., Burdick J.A. (2013). The influence of hyaluronic acid hydrogel crosslinking density and macromolecular diffusivity on human MSC chondrogenesis and hypertrophy. Biomaterials.

[18] Bartnikowski M., Bartnikowski N., Woodruff M., Schrobback K., Klein T. (2015). Protective effects of reactive functional groups on chondrocytes in photocrosslinkable hydrogel systems. Acta Biomater..

[19] Li X., Chen Y., Kawazoe N., Chen G. (2017). Influence of microporous gelatin hydrogels on chondrocyte functions. J. Mater. Chem. B.

[20] Reis A.V., Fajardo A.R., Schuquel I.T., Guilherme M.R., Vidotti G.J., Rubira A.F., Muniz E.C. (2009). Reaction of glycidyl methacrylate at the hydroxyl and carboxylic groups of poly (vinyl alcohol) and poly (acrylic acid): Is this reaction mechanism still unclear?. J. Org. Chem..

[21] Hoch E., Hirth T., Tovar G.E., Borchers K. (2013). Chemical tailoring of gelatin to adjust its chemical and physical properties for functional bioprinting. J. Mater. Chem. B.

[22] Li X., Li B., Ma J., Wang X., Zhang S. (2014). Development of a silk fibroin/HTCC/PVA sponge for chronic wound dressing. J. Bioact. Comp. Polym..

[23] Chen S., Zhang Q., Nakamoto T., Kawazoe N., Chen G. (2016). Gelatin scaffolds with controlled pore structure and mechanical property for cartilage tissue engineering. Tissue Eng. C.

[24] Cai R., Nakamoto T., Kawazoe N., Chen G. (2015). Influence of stepwise chondrogenesis-mimicking 3D extracellular matrix on chondrogenic differentiation of mesenchymal stem cells. Biomaterials.

[25] Lu H., Ko Y.-G., Kawazoe N., Chen G. (2010). Cartilage tissue engineering using funnel-like collagen sponges prepared with embossing ice particulate templates. Biomaterials.

[26] Chen Y.C., Lin R.Z., Qi H., Yang Y., Bae H., Melero-Martin J.M., Khademhosseini A. (2012). Functional human vascular network generated in photocrosslinkable gelatin methacrylate hydrogels. Adv. Funct. Mater..

[27] Chen S., Zhang Q., Kawazoe N., Chen G. (2015). Effect of high molecular weight hyaluronic acid on chondrocytes cultured in collagen/hyaluronic acid porous scaffolds. RSC Adv..

[28] Fan C., Wang D.A. (2015). Effects of permeability and living space on cell fate and neo-tissue development in hydrogel-based scaffolds: A study with cartilaginous model. Macromol. Biosci..

[29] Kim I.L., Khetan S., Baker B.M., Chen C.S., Burdick J.A. (2013). Fibrous hyaluronic acid hydrogels that direct MSC chondrogenesis through mechanical and adhesive cues. Biomaterials.

[30] Moreira Teixeira L., Leijten J., Sobral J., Jin R., Apeldoorn A., Feijen J., Blitterswijk C., Dijkstra P., Karperien H. (2012). High throughput generated micro-aggregates of chondrocytes stimulate cartilage formation in vitro and in vivo. Eur. Cells Mater..

[31] Fang J., Yong Q., Zhang K., Sun W., Yan S., Cui L., Yin J. (2015). Novel injectable porous poly (γ-benzyl-l-glutamate) microspheres for cartilage tissue engineering: Preparation and evaluation. J. Mater. Chem. B.

[32] Bryant S.J., Durand K.L., Anseth K.S. (2003). Manipulations in hydrogel chemistry control photoencapsulated chondrocyte behavior and their extracellular matrix production. J. Biomed. Mater. Res. A.

[33] Gosset M., Berenbaum F., Thirion S., Jacques C. (2008). Primary culture and phenotyping of murine chondrocytes. Nat. Protoc..

[34] Stewart M.C., Saunders K.M., Burton-Wurster N., Macleod J.N. (2000). Phenotypic stability of articular chondrocytes in vitro: The effects of culture models, bone morphogenetic protein 2, and serum supplementation. J. Bone Miner. Res..

[35] Sung L.-Y., Lo W.-H., Chiu H.-Y., Chen H.-C., Chung C.-K., Lee H.-P., Hu Y.-C. (2007). Modulation of chondrocyte phenotype via baculovirus-mediated growth factor expression. Biomaterials.

[36] De Crombrugghe B., Lefebvre V., Behringer R.R., Bi W., Murakami S., Huang W. (2000). Transcriptional mechanisms of chondrocyte differentiation. Matrix Biol..

[37] Lefrebvre V., de Crombrugghe B. (1998). Toward understanding SOX9 function in chondrocyte differentiation. Matrix Biol..

[38] Shin S.R., Aghaei-Ghareh-Bolagh B., Dang T.T., Topkaya S.N., Gao X., Yang S.Y., Jung S.M., Oh J.H., Dokmeci M.R., Tang X.S. (2013). Cell-laden microengineered and mechanically tunable hybrid hydrogels of gelatin and graphene oxide. Adv. Mater..

[39] Miao T., Miller E.J., McKenzie C., Oldinski R.A. (2015). Physically crosslinked polyvinyl alcohol and gelatin interpenetrating polymer network theta-gels for cartilage regeneration. J. Mater. Chem. B.

[40] Zhang J., Li J., Chen S., Kawazoe N., Chen G. (2016). Preparation of gelatin/Fe_3_O_4_ composite scaffolds for enhanced and repeatable cancer cell ablation. J. Mater. Chem. B.

[41] Zhang J., Li J., Kawazoe N., Chen G. (2017). Composite scaffolds of gelatin and gold nanoparticles with tunable size and shape for photothermal cancer therapy. J. Mater. Chem. B.

[42] Truong V.X., Hun M.L., Li F., Chidgey A.P., Forsythe J.S. (2016). In situ-forming click-crosslinked gelatin based hydrogels for 3D culture of thymic epithelial cells. Biomater. Sci..

[43] Wissink M., Beernink R., Pieper J., Poot A.A., Engbers G., Beugeling T., Van Aken W., Feijen J. (2001). Immobilization of heparin to EDC/NHS-crosslinked collagen. Characterization and in vitro evaluation. Biomaterials.

[44] Martucci J., Accareddu A., Ruseckaite R. (2012). Preparation and characterization of plasticized gelatin films cross-linked with low concentrations of glutaraldehyde. J. Mater. Sci..

[45] Yan L.P., Wang Y.J., Ren L., Wu G., Caridade S.G., Fan J.B., Wang L.Y., Ji P.H., Oliveira J.M., Oliveira J.T. (2010). Genipin-cross-linked collagen/chitosan biomimetic scaffolds for articular cartilage tissue engineering applications. J. Biomed. Mater. Res. A.

[46] Zhang X., Xu L., Huang X., Wei S., Zhai M. (2012). Structural study and preliminary biological evaluation on the collagen hydrogel crosslinked by γ-irradiation. J. Biomed. Mater. Res. A.

[47] Higuchi A., Ling Q.-D., Kumar S.S., Chang Y., Alarfaj A.A., Munusamy M.A., Murugan K., Hsu S.-T., Umezawa A. (2015). Physical cues of cell culture materials lead the direction of differentiation lineages of pluripotent stem cells. J. Mater. Chem. B.

[48] Wang X., Nakamoto T., Dulińska-Molak I., Kawazoe N., Chen G. (2016). Regulating the stemness of mesenchymal stem cells by tuning micropattern features. J. Mater. Chem. B.

